# Cyber Risk Propagation and Optimal Selection of Cybersecurity Controls for Complex Cyberphysical Systems

**DOI:** 10.3390/s21051691

**Published:** 2021-03-01

**Authors:** Georgios Kavallieratos, Georgios Spathoulas, Sokratis Katsikas

**Affiliations:** Department of Information Security and Communication Technology, Norwegian University of Science and Technology, N-2815 Gjøvik, Norway; georgios.kavallieratos@ntnu.no (G.K.); georgios.spathoulas@ntnu.no (G.S.)

**Keywords:** cybersecurity, cyber physical systems, cyber risk propagation, cybersecurity controls, autonomous vessels

## Abstract

The increasingly witnessed integration of information technology with operational technology leads to the formation of Cyber-Physical Systems (CPSs) that intertwine physical and cyber components and connect to each other to form systems-of-systems. This interconnection enables the offering of functionality beyond the combined offering of each individual component, but at the same time increases the cyber risk of the overall system, as such risk propagates between and aggregates at component systems. The complexity of the resulting systems-of-systems in many cases leads to difficulty in analyzing cyber risk. Additionally, the selection of cybersecurity controls that will effectively and efficiently treat the cyber risk is commonly performed manually, or at best with limited automated decision support. In this work, we propose a method for analyzing risk propagation and aggregation in complex CPSs utilizing the results of risk assessments of their individual constituents. Additionally, we propose a method employing evolutionary programming for automating the selection of an optimal set of cybersecurity controls out of a list of available controls, that will minimize the residual risk and the cost associated with the implementation of these measures. We illustrate the workings of the proposed methods by applying them to the navigational systems of two variants of the Cyber-Enabled Ship (C-ES), namely the autonomous ship and the remotely controlled ship. The results are sets of cybersecurity controls applied to those components of the overall system that have been identified in previous studies as the most vulnerable ones; such controls minimize the residual risk, while also minimizing the cost of implementation.

## 1. Introduction

Cyber-Physical Systems (CPSs) are characterized by the strong coupling of the physical and the cyber worlds. The inevitable dependence on highly automated procedures and the increasing integration of *physical parts* to highly interconnected *cyber parts* render CPSs vulnerable to cyber attacks. On the other hand, the wide use of such systems in various critical domains [1] (e.g., Smart Grid, Intelligent Transportation Systems, Medical devices, Industrial Control Systems, etc.) increases the impact of such cyber attacks. Furthermore, the *System of Systems* (SoS) nature of interconnected, complex CPSs [2] introduces challenges in addressing security risks. In this context, a complex CPS comprises other CPSs that are interconnected, and control and information flows exist among them. These flows constitute pathways that a cyber attack may leverage to propagate from component to component. More specifically, both or either of the likelihood of the attack and its impact, if successful, may propagate. Because likelihood and impact are the constituents of risk, the cyber risk of the overall system is related to the individual cyber risk of each interconnected component. This in principle means that knowledge of the cyber risk of the individual components of a complex CPS may be leveraged to assess the cyber risk of the overall system, thus also facilitating the analysis of large scale, complex CPSs through a divide-and-conquer-like approach to cyber risk assessment.

The assessment of risk is one of the steps in the risk management process [3] that concludes with treating the risk by means of controls that aim at achieving retention, reduction, transfer, or avoidance of the risk [4]. In the general case, each risk can be treated by a number of possible cybersecurity controls, each of which with varying effectiveness and efficiency characteristics. Note that the same control may be effective and efficient in treating more than one risk. Therefore, an important task in formulating the risk treatment plan is the selection of the optimal set of cybersecurity controls, the criterion of optimality in this context being effectiveness and efficiency. Because of the complexity of formulating this as a formal optimization problem, particularly when there are more than one criteria of optimality, the selection of the cybersecurity controls is largely performed empirically, at best with some automated decision support.

In this paper, we propose a novel method for identifying a set of effective and efficient cybersecurity controls for large scale, complex CPSs comprising other CPSs as components. We also propose a method for assessing the aggregated risk that results by taking into account the risk of the individual components and the information and control flows among these components. Specifically, we leverage evolutionary computing to develop a cybersecurity control selection algorithm that uses the aggregated cyber risk of a complex CPS to generate a set of effective and efficient cybersecurity controls to reduce this risk. The algorithm selects the cybersecurity controls among the list of such controls in the NIST Guidelines for Industrial Control Systems Security [5]. We illustrate the workings of the proposed method by applying it to the navigational systems of two instances of the Cyber-Enabled Ship (C-ES), i.e., vessels with enhanced monitoring, communication, and connection capabilities that include remotely controlled and fully autonomous ships [6]. The C-ES comprises a variety of interconnected and interdependent CPSs [7], and, as such, it constitutes a complex CPS. Specifically, we derive the set of cybersecurity controls for both the autonomous and the remotely controlled vessel.

Thus, the contribution of this work is as follows:

- A novel method for assessing the aggregate cybersecurity risk of a large scale, complex CPS comprising components connected via links that implement both information and control flows, by using risk measures of its individual components and the information and control flows among these components.
- A novel method for selecting a set of effective and efficient cybersecurity controls among those in an established knowledge base, that reduce the residual risk, while at the same time minimizing the cost.
- Sets of cybersecurity controls for the navigational systems of two instances of the C-ES, namely the remotely controlled ship and the autonomous ship, derived by employing the two methods.

The remainder of this paper is structured as follows: Section 2 reviews the related work in the areas of cyber risk propagation and aggregation; optimal selection of cybersecurity controls; and C-ES risk management. Section 3 provides the background knowledge on genetic algorithms, and on the STRIDE (*S*poofing, *T*ampering, *R*epudiation, *I*nformation disclosure, *D*enial of Service, and *E*levation) and DREAD (*D*amage, *R*eproducibility, *E*xploitability, *A*ffected, and *D*iscoverability) risk assessment methods that is necessary to make the paper self-sustained. Section 4 and Section 5 present the proposed method for risk aggregation in complex CPSs and the proposed method for optimal cybersecurity control selection, respectively. In Section 6, we apply the proposed methods to the remotely controlled and the autonomous ship cases and discuss the results. Finally, Section 7 summarizes our conclusions and outlines topics for future research work.

## 2. Related Work

Cyber risk is evaluated as a function of the likelihood of an adverse event, such as an attack, occurring; and of the impact that will result when the event occurs. In order for an adverse event to occur, a threat has to successfully exploit one or more vulnerabilities; this can be done by launching one of a number of possible attacks. Hence, the likelihood of the event occurring is, in turn, determined by the likelihood of the threat successfully exploiting at least one vulnerability. Accordingly, in order to analyze how the cyber risk propagates in a complex system made up by interconnected components that are systems by themselves requires analyzing how both the likelihood of the event and its impact propagates. Once this analysis is accomplished, the aggregate cyber risk of the complex system can be assessed.

Several security risk assessment methods applicable to general purpose IT systems have appeared in the literature (see Reference [8] for a comprehensive survey). Even though several of these methods can be and have been applied to CPSs, they cannot accurately assess cyber risks related to CPSs according to Reference [9], where a number of approaches for risk assessment for CPSs are listed. A review of risk assessment methods for CPSs, from the perspective of safety, security, and their integration, including a proposal for some classification criteria was made in Reference [10]. A survey of IoT-enabled cyberattacks that includes a part focused on CPS-based environments can be found in Reference [11]. Cyber risk assessment methods for CPSs more often than not are domain specific, as they need to take into account safety as an impact factor additional to the “traditional” impact factors of confidentiality, integrity, and availability. For example, an overview of such methods specific to the smart grid case is provided in Reference [12]. A review of the traditional cybersecurity risk assessment methods that have been used in the maritime domain, is provided in Reference [13]. Additionally, various risk assessment methods have been proposed to analyze cyber risk in autonomous vessels [14,15,16].

Several works in the literature have studied how individual elements of cyber risk propagate in a network of interconnected systems; both deterministic and stochastic approaches have been used to this end. A threat likelihood propagation model for information systems based on the Markov process was proposed in Reference [17]. An approach for determining the propagation of the design faults of an information system by means of a probabilistic method was proposed in Reference [18]. A security risk analysis model (SRAM) that allows the analysis of the propagation of vulnerabilities in information systems, based on a Bayesian network, was proposed in Reference [19]. Methods for evaluating the propagation of the impact of cyber attacks in CPSs have been proposed in References [20,21,22], among others. Epidemic models were initially used to study malware propagation in information systems [17]. The propagation of cybersecurity incidents in a CPS is viewed as an epidemic outbreak in Reference [23] and is analyzed using percolation theory. The method was shown to be applicable for studying malware infection incidents, but it is questionable whether the epidemic outbreak model fits other types of incidents. Percolation theory was also used in Reference [24] to analyze the propagation of node failures in a network of CPSs comprising cyber and physical nodes organized in two distinct layers, such as in the case of the power grid. The Susceptible–Exposed–Infected–Recovered (SEIR) infectious disease model was used in Reference [25] to study malware infection propagation in the smart grid. A quantitative risk assessment model that provides asset-wise and overall risks for a given CPS and also considers risk propagation among dependent nodes was proposed in Reference [26].

A method for assessing the aggregate risk of a set of interdependent critical infrastructures was proposed in References [27,28]. The method provides an aggregate cyber risk value at the infrastructure level, rather than a detailed cyber risk assessment at the system/component level. Thus, it is suitable for evaluating the criticality of infrastructure sectors, but not for designing cybersecurity architectures or for selecting appropriate cybersecurity controls. A similar approach for the Energy Internet [29] was followed to develop an information security risk algorithm based on dynamic risk propagation in Reference [30]. A framework for modeling and evaluating the aggregate risk of user activity patterns in social networks was proposed in Reference [31]. A two-level hierarchical model was used in Reference [32] to represent the structure of essential services in the national cyberspace, and to evaluate the national level (aggregate) risk assessment by taking into account cyber threats and vulnerabilities identified at the lower level.

Based on the above discussion, it is evident that the problem of risk propagation and risk aggregation for complex systems, on one hand, and the problem of optimal selection of cybersecurity controls, on the other, have been individually studied. The conjunct problem of identifying the optimal set of cybersecurity controls that reduces the aggregate risk in a complex CPS cannot be approached by sequential application of methods each of which addresses the problem’s components, due to the inherent nonlinearity of the risk propagation, risk aggregation, and control selection processes on one hand, and the intertwining of these processes. To the best of our knowledge, no method that solves this conjunct problem is currently available.

On the other hand, the systematic selection of cybersecurity controls has been mostly examined in the literature in attempting to identify the optimal set of controls for IT systems within a specified budget; examples of such approaches are those in References [33,34,35]. The outline of a programming tool that supports the selection of countermeasures to secure an infrastructure represented as a hierarchy of components was provided in Reference [36]. A methodology based on an attack surface model to identify the countermeasures against multiple cyberattacks that optimize the Return On Response Investment (RORI) measure is proposed in Reference [37]. However, to the best of our knowledge, a method that selects a set of cybersecurity controls that simultaneously optimizes both effectiveness and efficiency, by minimizing the residual risk and the cost of implementation, is still to be proposed.

The work described in this paper addresses these research gaps.

## 3. Background

### 3.1. Evolutionary/Genetic Algorithms

Genetic algorithms (GAs) are randomized search algorithms that imitate the structures of natural genetics and the mechanisms of natural selection [38]. They imitate biological genomes by means of strings structures that represent individuals and are composed of characters belonging to a sepcified alphabet. These structures form populations that evolve in time by means of a randomized exchange scheme that implements the principle of survival of the fittest; in every new generation, a new set of individuals is created, using parts of the fittest members of the old set, whilst also possibly retaining some of the fittest members of the old generation. GAs can be very useful when it comes to problems with very large solution spaces, where it is infeasible to exhaustively search the solution space. It should, however, be noted that GAs are not guaranteed to find the global optimum solution to a problem; however, they do find “acceptably good” solutions.

For designing a GA, a *coding scheme* that codes the parameter space; a set of *operators* to be used to each generation to generate the next generation; and a *fitness function* that measures the fitness of each individual as a functional of the function that we are trying to optimize need to be defined. The coding scheme and the fitness function to be used depend on the characteristics of the optimization problem on which the GA will be applied. However, a commonly used coding scheme is to use the binary alphabet to represent each element (gene) in a string (genome). On the other hand, the most commonly used operators are the *reproduction* operator, the *crossover* operator, and the *mutation* operator. These have been found to be both computationally simple and effective in a number of optimization problems [39].

The operators are used to evolve populations by creating new individuals that will form the new generation. To this end, the reproduction operator tentatively selects individuals with high fitness function values as candidate parents for the next generation, by means of a randomized technique, such as a *roulette wheel selection scheme*. The selected parents may mate by means of the crossover operator, that randomly selects pairs of mates and creates new individuals, by combining elements of both parents, these elements being selected at random. As in biological populations, random genetic alterations (mutations) sometimes result in genetically fitter individuals. Such alterations, that happen with small probability, are implemented in GAs by means of the mutation operator.

The generic GA addresses unconstrained optimization problems. However, constrained optimization problems are encountered more often than not, including the problem addressed in this work, as will be seen in the sequel. Constraints can be modeled as either equality relations, that can be incorporated within the function to be optimized; or as inequality relations, that may be handled either by simply evaluating the fitness of each individual and then check to see whether any constraints are violated, or by employing a penalty method. In the former (reactive) strategy, if an individual violates a constraint, it is assigned a fitness value equal to zero. In the latter (proactive) strategy, the fitness of an individual that violates a constraint is decreased by an amount proportional to the cost of the violation.

### 3.2. STRIDE

STRIDE [40] is a cyber security threat modeling method that was developed at Microsoft in 1999. It facilitates the process of identifying and analyzing six types of threats, namely *S*poofing, *T*ampering, *R*epudiation, *I*nformation disclosure, *D*enial of Service, and *E*levation of privileges, in which the initials form the acronym *STRIDE*. Each of these threats corresponds to the violation of a desirable property (security objective) of the system under study, as follows:

- Spoofing corresponds to violation of authenticity;
- Tampering corresponds to violation of integrity;
- Repudiation corresponds to violation of non-repudiability;
- Information disclosure corresponds to violation of confidentiality;
- Denial of service corresponds to violation of availability; and
- Elevation of privileges corresponds to violation of authorization.

STRIDE can be used to analyze threats for systems being in a variety of development phases, even for systems at the design phase; thus, it enables adherence to security-by-design principles [41]. Furthermore, even though originally designed for software systems, STRIDE has been also used in ecosystem environments where CPSs are prominently present [42,43,44]. In particular, a modified version of STRIDE was proposed and used in Reference [6] to model threats, to develop cyber attack scenarios, and to qualitatively assess the accordant risks for a number of CPSs in the C-ES ecosystem.

### 3.3. DREAD

DREAD is a security risk assessment model that, like STRIDE, was developed as part of Microsoft’s threat modeling and risk analysis process. The name is an acronym made up from the initials of the characteristics of the risk associated with each attack scenario being analyzed, namely *D*amage (what is the extent of the damage that the attack is expected to inflict on the system); *R*eproducibility (how easy it is to reproduce the attack); *E*xploitability (the extent of the resources that the adversary needs to launch the attack); *A*ffected users/systems (how many people and/or systems will be affected); and *D*iscoverability (how easy is it for the adversary to identify vulnerabilities to exploit for launching the attack) [45].

STRIDE and DREAD are interrelated: the former allows the qualitative security analysis of the system, whilst the latter quantifies the identified risks. According to the approach in Reference [22], the values (High, Medium, Low) of the DREAD variables associated with each STRIDE threat t∈{S,T,R,I,D,E} are determined by applying a specific set of criteria, shown in Table 1; these have been adapted from those in Reference [45], so as to include CPS aspects, and are further analyzed in Reference [22].

Then, the risk value $R_{t}^{s}$ associated with each STRIDE threat t∈{S,T,R,I,D,E} for system *s* is calculated by using the following formulas [41,44,45]:

(1) $$Impact_{t}^{s}=\frac{Damage+Affectedsystems}{2},$$

(2) $$Likelihood_{t}^{s}=\frac{Reproducibility+Exploitability+Discoverability}{3},$$

(3) $$Risk_{t}^{s}=\frac{\left(Impact_{t}^{s}+Likelihood_{t}^{s}\right)}{2}.$$

$Impact_{t}^{s}$ represents a measure of the effect a successful attack materializing threat *t* has on the component *s*; $Likelihood_{t}^{s}$ represents a measure of how likely it is for threat *t* to materialize on *s*.

Both STRIDE and DREAD have been used in Reference [44] to assess the cyber risk of Cyber-Physical Systems (CPSs) on board the C-ES paradigm.

## 4. Cyber Risk Propagation and Aggregation

### 4.1. System Model

**Figure 1:**
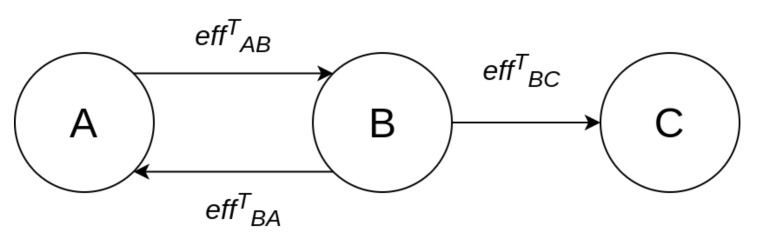
Effect relationship between nodes.

Assume a CPS consisting of *N* interconnected components, each denoted by $c_{i},\;i=1,...N$. This system can be represented by a directed graph of N+1 nodes, the system itself being one of the nodes, denoted as $c_{0}$. The edges of the graph represent information and control flows between the nodes. An edge from node *A* to node *B* indicates the existence of either an information flow or a control flow, from *A* to *B*. A consequence of the existence of such an edge is that a cybersecurity event at node *A* affects node *B*, as well. For example, in the simple graph of Figure 1, a cybersecurity event at node *A* will have effect on node *B*, as well, while a cybersecurity event at node *B* will have effect on both nodes *A* and *C*. The relationship "has effect" can be quantified by assigning an *effect coefficient* to each flow. These are denoted henceforth by $eff_{AB}^{a}$, where a=I for the information flow, and a=C for the control flow, respectively. One way of assigning values to these coefficients is to use the inverse of the *in degree centrality*, i.e., the number of flows arriving to that node, denoted by IDC. Following this approach, the case in which information arrives to node *B* only through node *A*, will result in a much higher $eff_{AB}^{I}$ than the case where information arrives to node *B* from a large number of nodes, including *A*. By definition, the values of all effect coefficients lie in the [0, 1] range and provide an indication of the percentage of the damage that is propagated from one node to the other. The *total effect coefficient* $eff_{AB}^{T}$ is computed as a function of $eff_{AB}^{I}$ and $eff_{AB}^{C}$, as in Equation (4).

The function *f* in Equation (4) has to be instantiated according to the requirements of the domain to which the methodology is applied and/or to specific characteristics of components *A* and *B* with regards to the criticality of information and control flows between them. For example, one option is to select *f* as the average of the effect coefficients. This option reflects equal importance of the information and the control flows in risk propagation, and it has been used in the illustrative application of the method presented in Section 6.

(4) $$eff_{AB}^{T}=f\left(eff_{AB}^{I},eff_{AB}^{C}\right),$$

where $eff_{AB}^{I}=\frac{1}{IDC_{B}^{I}},\;\;eff_{AB}^{C}=\frac{1}{IDC_{B}^{C}}$.

Another example is that of a cyber-physical system that mainly aims at sensing and processing data coming from a process, e.g., an electric power smart meter. In such systems, information workflows are more significant than control flows, and a function *f* of the form $eff_{AB}^{T}=a\times eff_{AB}^{I}+b\times eff_{AB}^{C}$ with a+b=1,a>b would be a good choice. On the other hand, for a cyber-physical system that aims at controlling a process, e.g., a smart grid digital switch, a variant of the same function *f* but with a+b=1,b>a would be more appropriate, as control flows are more likely to enable cyber risk propagation between components.

### 4.2. Aggregate Risk

For any threat *t*, the *aggregate* risk $R_{t}^{agg_{c_{j}}}$ of component $c_{j}$ is (applying the worst case scenario principle [28]) given by:

(5) $$R_{t}^{agg_{c_{j}}}=\max\left(R_{t}^{dir_{c_{j}}},R_{t}^{prop_{c_{j}}}\right),$$

where $R_{t}^{dir_{c_{j}}}$ (*direct* risk) is the risk when $c_{j}$ is not connected to any other component $c_{k},\;k\neq j$, which is calculated by means of Equations (1)–(3), and $R_{t}^{prop_{c_{j}}}$ (*propagated* risk) is the risk that $c_{j}$ faces because of its connections to other components. These connections may be over any, possibly multi-hop, path $p_{l}$ from any node *k* to *j*, k≠j. Applying again the worst case scenario principle, $R_{t}^{prop_{c_{j}}}$ is calculated as:

(6) $$R_{t}^{prop_{c_{j}}}=\max_{p_{l}}R_{t}^{prop_{c_{j}}^{p_{l}}},$$

where $R_{t}^{prop_{c_{j}}^{p_{l}}}$ is the risk of component $c_{j}$ associated with threat *t* and propagated along path $p_{l}$.

When a threat materializes against component $c_{i}$, it will also create an effect to component $c_{j}$, if $c_{i}$ and $c_{j}$ are connected. In the absence of controls, the likelihood that this will happen is equal to the likelihood that the threat will materialize against $c_{i}$ in the first place. In contrast, the impact that this event has on $c_{j}$ is only a fraction of the impact the event has on any $c_{k}$ on any path $p_{l}$ from $c_{i}$ to $c_{j}$. This fraction is represented by $eff_{p_{l}}^{T}$ and is calculated by

(7) $$eff_{p_{l}}^{T}=\prod_{i=1}^{j-1}eff_{c_{i}c_{i+1}}^{T}.$$

Accordingly, the risk propagated over path $p_{l}$, originating at component (node) $c_{i}$ and terminating at component (node) $c_{j}$, is calculated by:

(8) $$R_{t}^{prop_{c_{j}}^{p_{l}}}=\frac{eff_{c_{i}c_{j}}^{T_{p_{l}}}\times Impact_{t}^{c_{i}}+L_{t}^{c_{i}}}{2}.$$

The system as a whole is represented by $c_{0}$; therefore, the (global) risk of threat *t* for the system is given by:

(9) $$R_{t}^{s}=R_{t}^{agg_{c_{0}}}=\max\left(R_{t}^{dir_{c_{0}}},R_{t}^{prop_{c_{0}}}\right),$$

where the direct risk for the system is not applicable ($R_{t}^{dir_{c_{0}}}=0$) and the propagated risk for the system is calculated as for any other node ($R_{t}^{prop_{c_{0}}}=\max_{p_{l}}R_{t}^{prop_{c_{0}}^{p_{l}}}$), thus

(10) $$R_{t}^{s}=\max_{p_{l}}R_{t}^{prop_{c_{0}}^{p_{l}}}$$

**Figure 2:**
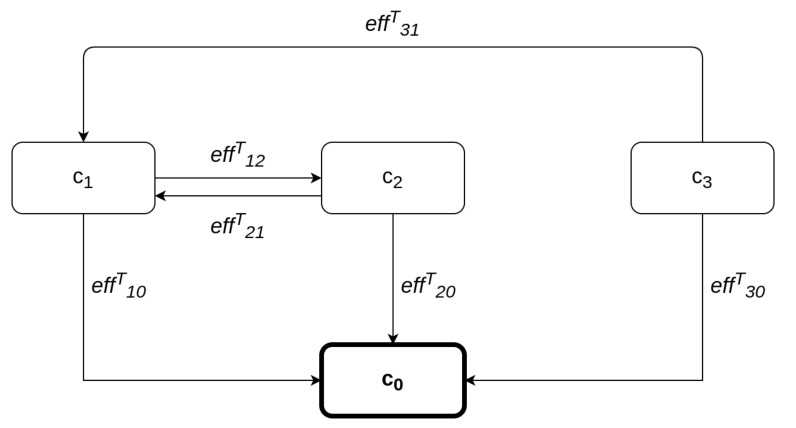
An example of a system.

In order to showcase how the global risk calculation works and also to shed light on an underlying subtle assumption, consider the example system shown in Figure 2. In order to calculate the aggregate risk of each $c_{i}$, i=1,2,3, we need to calculate the propagated risks, and this requires identifying all possible paths originating at any node and terminating at $c_{i}$, i=1,2,3, respectively. The propagated risk for $c_{3}$ is equal to zero, as there is no such path. Nodes $c_{1}$ and $c_{2}$ are interconnected; therefore, a loop exists between them. Consequently, if we allow circular paths to be considered, there are infinite paths between these two nodes, and the computation in Equation (7) would be endless. However, by noticing that the value of the total effect coefficient becomes, by definition, negligible after a couple of hops, we are able to disregard circular paths in its computation.

Therefore, the global risk of a system can be calculated by the algorithm in Algorithm 1. As can be seen in Algorithm 1, nodes along a path are processed recursively, starting at the end of the path. If a node is already in the path, it is not included again, so as to avoid cyclic paths.
 **Algorithm 1:** Global system risk calculation algorithm. 
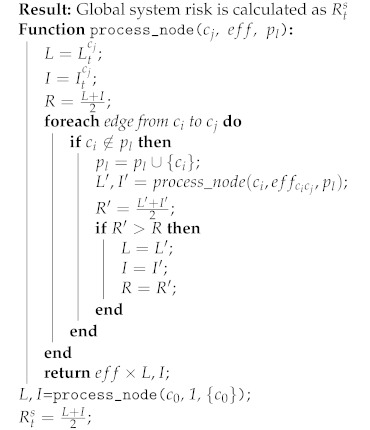


## 5. Optimal Cybersecurity Control Selection

### 5.1. Cybersecurity Controls

We assume that there exists a list of controls available to apply to the components of the system. Each control *m*, when applied to component $c_{i}$, has a potential effect on the values of $Impact_{t}^{c_{i}}$ and $Likelihood_{t}^{c_{i}}$ that are used in the calculation of the cyber risk, such effect depending on the effectiveness and the nature of the control. We denote the new Likelihood and Impact values of threat *t* that result after the application of control *m* to $c_{i}$ by $Likelihood_{t^{m}}^{c_{i}}$ and $Impact_{t^{m}}^{c_{i}}$, respectively. These values can be calculated by re-applying DREAD to the system, which is now protected by *m*.

Additionally, for each control *m*, a cost metric $Cost_{m}$ is defined. This metric is expressed on a 1–5 scale, corresponding to the qualitative classifications very low cost, low cost, medium cost, high cost, and very high cost. Note that the use of this scale was dictated by the fact that it is difficult to measure the cost of implementing a control. However, if such a measure is available, the replacement of the value in the 1–5 scale with the actual cost of the control is straightforward.

For a system with *N* components and a list with *M* controls with the cost metrics vector $C=[cost_{1},cost_{2},\ldots ,cost_{M}]$, the following binary matrix AC compactly depicts the applied controls throughout the system:

(11) $$AC=\left[\begin{array}{cccc} ac_{1,1} & ac_{1,2} & \ldots & ac_{1,N}\\ ac_{2,1} & ac_{2,2} & \ldots & ac_{2,N}\\ \ldots & \ldots & \ldots & \ldots \\ ac_{M,1} & ac_{M,2} & \ldots & ac_{M,N} \end{array}\right],$$

where

(12) $$ac_{i,j}=\left\{\begin{array}{c} 0,\mathrm{if}\mathrm{control}i\mathrm{is}\mathrm{not}\mathrm{applied}\mathrm{to}\mathrm{component}j\\ 1,\mathrm{if}\mathrm{control}i\mathrm{is}\mathrm{applied}\mathrm{to}\mathrm{component}j \end{array}\right..$$

Then, the total cost $TC_{AC}$ of the applied controls solution AC is given by $TC_{AC}=AC\times C$.

### 5.2. Optimization Method

The optimization problem to be solved is to select the optimal (effective and efficient) set of controls among a list of possible ones. This amounts to selecting the set of controls AC that minimizes the system *residual* risk $R_{t_{AC}}^{s}$, at the lowest total cost TC. A closed formula that would allow the application of an exact optimization method, and thus the calculation of the globally optimum solution to the problem, is not possible to construct, unless many, not necessarily realistic, assumptions are made. On the other hand, the large size of the search space (all candidate solutions) prohibits the exhaustive search approach. Hence, a heuristic optimization method has to be employed [46]; we have selected to use a genetic algorithm, even though any other heuristic optimization method would, in principle, be applicable.

The design parameters of the genetic algorithm are as follows:

- The search space comprises all possible combinations of controls applied to components.
- Each individual solution is represented by the matrix AC, which is transformed into a binary vector of size M×N. The value of each element of the vector represents the decision to apply a specific control to a specific component or not. For example, for a system with three components and two controls, the solution would be denoted by the vector $\left[ac_{11},ac_{21},ac_{12},ac_{22},ac_{13},ac_{23}\right]$, assuming that all controls are applicable to all components.
- The fitness function is defined as $fit\left(AC\right)=R_{t_{AC}}^{s}+C_{norm}\left(AC\right)$, where $C_{norm}\left(AC\right)=\frac{TC_{AC}}{TC_{max}}$, with $TC_{max}$ being the largest possible cost, that results when applying all available controls to all system components.
- The initial population size is 100.
- The mutation probability is 0.1.
- The next generation is determined by uniform crossover, with crossover probability equal to 0.5, an elite ratio of 0.01, and 0.3 of the population consisting of the fittest members of the previous generation (aka parents).
- The algorithm terminates when the maximum number of allowed iterations is used. This number is calculated as $iter_{max}=50\times \sum_{i=1,j=1}^{i=M,j=N}ac_{ij}$.

The algorithm for selecting the optimal set of security controls is depicted in Algorithm 2.

Note that the fitness function consists of two elements, namely the residual risk (which takes values in [0, 3]) and the normalized cost (which takes values in [0, 1]). This non-symmetric approach has been selected to put emphasis on the importance of reducing the residual risk, even by bearing larger cost. This approach results in initial iterations of the algorithm tending to generate solutions that minimize the residual risk. In later iterations of the algorithm, the less costly combinations of controls prevail, among those that lead to the maximum possible risk reduction.


 **Algorithm 2:** Algorithm for selecting the optimal set of security controls 
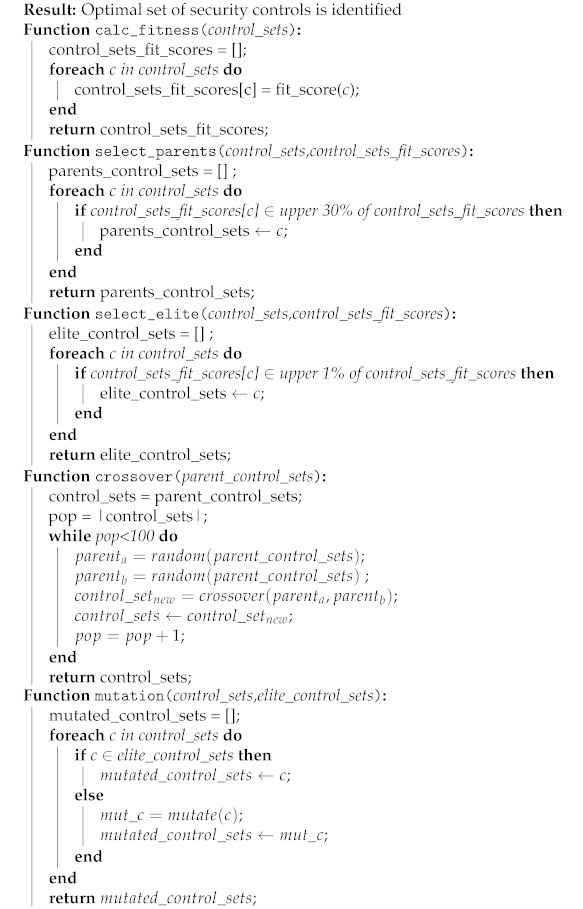
 
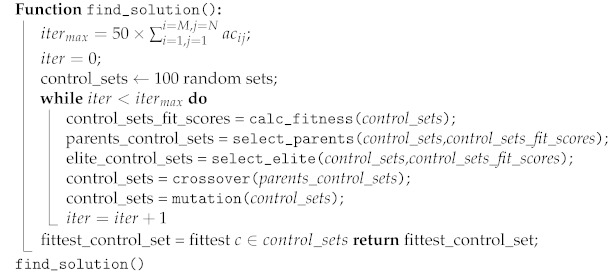



## 6. Application to the C-ES

**Figure 3:**
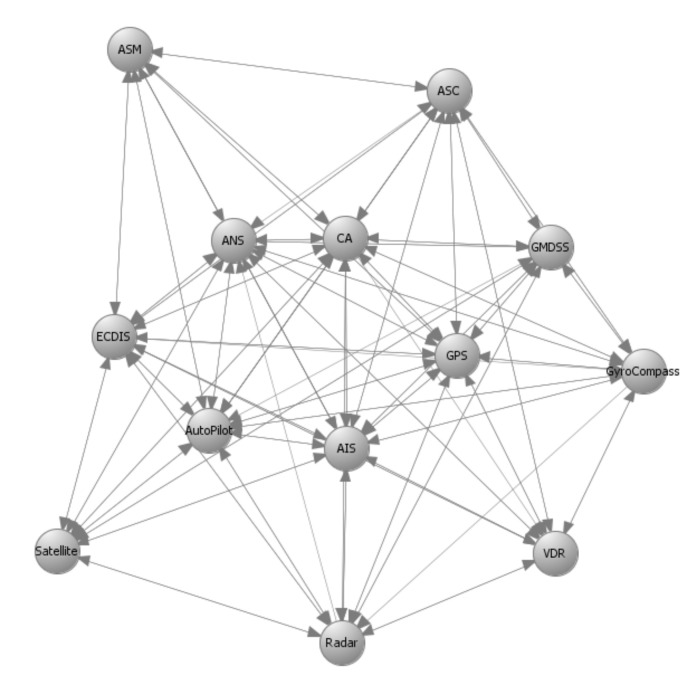
Autonomous ship—Navigational Cyber-Physical Systems (CPSs)–$G_{I}\left(V,E\right)$–Information flow connections.

**Figure 4:**
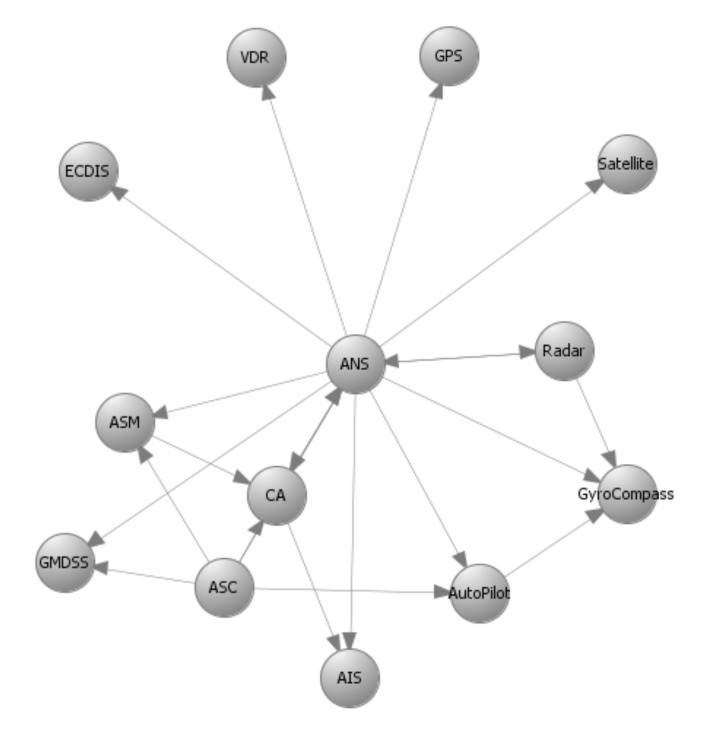
Autonomous ship—Navigational CPSs–$G_{C}\left(V,E\right)$–Control flow connections.

**Figure 5:**
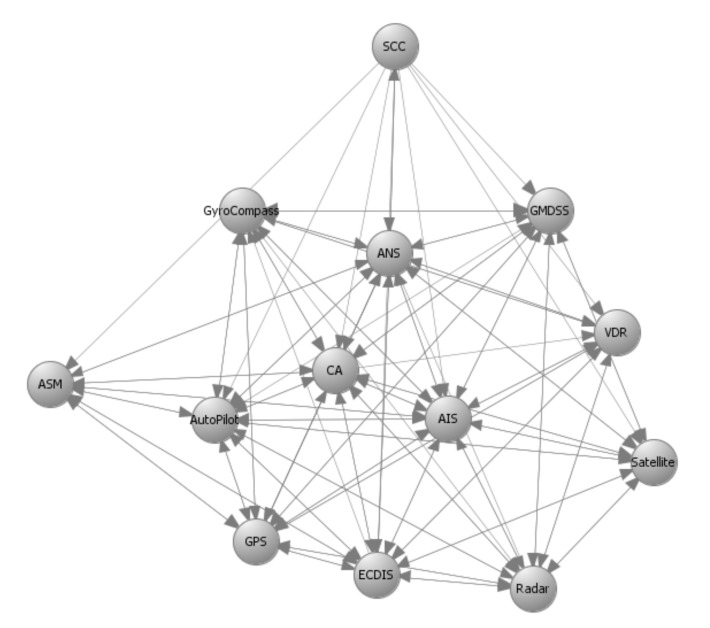
Remotely controlled ship—Navigational CPSs–$G_{I}^{\prime }\left(V,E\right)$–Information flows.

**Figure 6:**
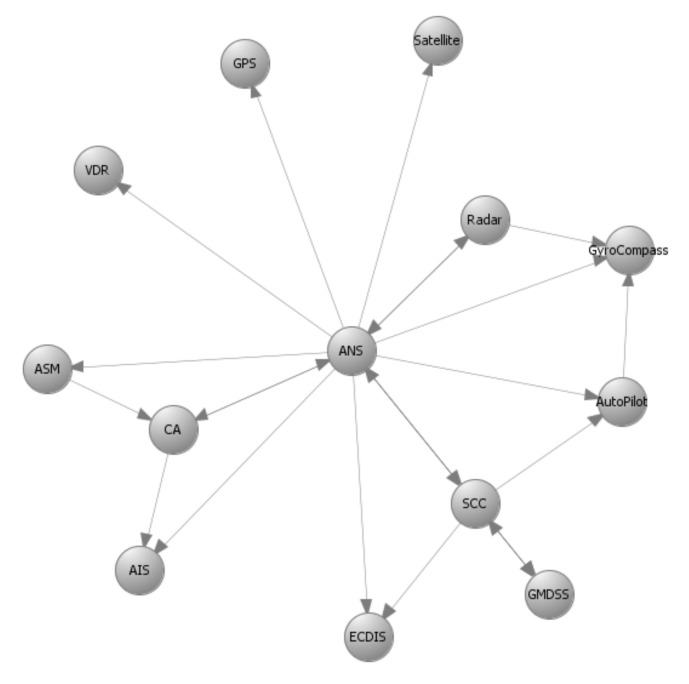
Remotely controlled ship—Navigational CPSs–$G_{C}^{\prime }\left(V,E\right)$–Control flows.

Autonomous and remotely controlled ships—both variants of the Cyber-Enabled Ship (C-ES)—are being increasingly developed. At the same time, the maritime transportation sector contributes significantly to the gross domestic product of many countries around the world. It is not surprising, then, that the cybersecurity of the sector has been designated a very high priority by international organizations [47] and national governments [48] alike. The CPSs comprising the C-ES were identified, and the overall ICT architecture of the C-ES in the form of a tree structure was proposed in Reference [6]. An extended Maritime Architectural Framework (e-MAF) was proposed, and the interconnections, dependencies, and interdependencies among the CPSs of the C-ES were described in Reference [7]. These results are depicted in the form of directed graphs in Figure 3, Figure 4, Figure 5 and Figure 6 for the two variants of the C-ES. Furthermore, an initial threat analysis of the generic ICT architecture of the C-ES identified the three most vulnerable onboard systems, namely the Automatic Identification System (AIS), the Electronic Chart Display Information System (ECDIS), and the Global Maritime Distress and Safety System (GMDSS) [6]. These results were verified by means of the comprehensive threat and risk analysis that was presented in Reference [44]. The most critical attack paths within the navigational CPSs of the C-ES were identified in Reference [22]. The cybersecurity and safety requirements for the CPSs of the C-ES were identified in References [49,50], and an initial set of cybersecurity controls that satisfy these requirements was proposed in Reference [44].

Building upon earlier work, and as a step towards defining the cybersecurity architecture of such vessels, we selected the CPSs of the C-ES to illustrate the applicability of the methods proposed in this paper. The results are presented in the sequel for the autonomous and the remotely controlled vessel.

### 6.1. The Cyber-Enabled Ship

The CPSs of the C-ES were identified and described in Reference [6], where a threat analysis and a qualitative risk analysis were carried out, and the most vulnerable onboard systems were identified. Three distinct sub-groups of onboard CPSs were identified, namely the bridge CPSs; the engine CPSs; and the Shore Control Center (SCC) CPSs. The SCC is a sub-component of the remotely controlled vessel, that aims to control and navigate one or more ships from the shore. The interconnections, dependencies, and interdependencies of these CPSs were identified in Reference [7] and were later used to define the cybersecurity requirements of the C-ES in Reference [49]. The CPSs considered herein are:

- The Autonomous Navigation System (ANS) is responsible for the navigational functions of the vessel. ANS controls all the navigational sub-systems and communicates with the SCC by transmitting dynamic, voyage, static, and safety data to ensure the vessel’s safe navigation.
- The Autonomous Ship Control (ASC) acts as an additional control for the C-ES and aims to assess the data derived from the sensors and from the SCC.
- The Advanced Sensor Module (ASM) automatically analyzes sensor data to enhance the environmental observations, such as ships in the vicinity. By leveraging sensor fusion techniques, this module analyzes data derived from navigational sensors, such as the Automatic Identification System (AIS) and the Radar.
- The Automatic Identification System (AIS) facilitates the identification, monitoring, and locating of the vessel by analyzing voyage, dynamic, and static data. Further, the AIS contributes to the vessel’s collision avoidance system by providing real time data.
- The Collision Avoidance (CA) system ensures the safe passage of the vessel by avoiding potential obstacles. The system analyzes the voyage path by leveraging anti-collision algorithms conforming to the accordant COLREGs regulations [51].
- The Electronic Chart Display Information System (ECDIS) supports the vessel’s navigation by providing the necessary nautical charts, along with vessel’s attributes, such as position and speed.
- The marine RADAR provides the bearing and distance of objects in the vicinity of the vessel, for collision avoidance and navigation at sea.
- The Voyage Data Recorder (VDR) gathers and stores all the navigational data of the vessel specifically related to vessel’s condition, position, movements, and communication recordings.
- The Auto Pilot (AP) controls the trajectory of the vessel without requiring continuous manual control by a human operator.

The methods proposed in Section 4 and Section 5 used as input prior results, namely the system components and their interconnections that make up the system graph representation; the impact and likelihood values associated with the STRIDE threats and computed by means of DREAD for each individual component; and the list of available cybersecurity controls, along with information on their cost and effectiveness. Figure 3, Figure 4, Figure 5 and Figure 6 depict the graph representations of the onboard navigational CPSs of the autonomous and of the remotely controlled ship, respectively, along with their interconnections and interdependencies [6,22,44]. Impact and likelihood values associated with the STRIDE threats and computed by means of DREAD are depicted in Table 2 and Table 3 [44]. Each line of Table 2 and Table 3 represents one of the STRIDE threats, indicated by the corresponding initial. Each column of the Table represents individual CPSs, indicated by their corresponding initials, as defined in Section 6.1. The values inside the cells are the corresponding impact (left table) and likelihood (right table) values per STRIDE threat and per individual component; these have been calculated by means of Equations (1) and (2), respectively. These values are subsequently used as input to Algorithm 1, to calculate the aggregate risk of each CPS.

The list of available cybersecurity controls has been defined based on the NIST guidelines for Industrial Control Systems security [5] by following a systematic process proposed in Reference [44]. The effectiveness and the cost of each security control are estimated considering their applicability, the extent to which each control reduces the impact or/and the likelihood, and the resources needed to implement it.

### 6.2. Optimal Controls for the Autonomous Ship

Autonomous ships are equipped with advanced interconnected CPSs able to navigate and sail the vessels without human intervention. The onboard navigational CPSs of the autonomous ship are described by the directed graphs $G_{I}\left(V,E\right)$ and $G_{C}\left(V,E\right)$ depicted in Figure 3 and Figure 4, respectively, as discussed in detail in References [6,44]. $G_{I}\left(V,E\right)$ represents information flow connections and $G_{C}\left(V,E\right)$ control flow connections. Table 4 depicts the effect coefficients between all the considered systems. Each line and each column of Table 4 represents a CPS of the C-ES, indicated by their corresponding initials, as defined in Section 6.1 above. The values inside the cells are the effect coefficients between each pair of these systems; specifically, the value in the cell at row *i* and column *j* is the value of $eff_{ij}^{T}$. These have been calculated by means of Equation (13), which derives from Equation (4) when the function *f* is the average of the information and control effect coefficients. These values are also subsequently used as input to Algorithm 1, to calculate the aggregate risk of each CPS.

(13) $$eff_{AB}^{T}=\frac{eff_{AB}^{I}+eff_{AB}^{C}}{2}.$$

It is worth noticing that CPSs with high information and control flows, such as the ANS and the ASC, are characterized by high values of the effect coefficient.

The security controls in the optimal set are selected from the initial list of available controls by applying the method described in Section 5. Table 5 depicts the optimal set of security controls per STRIDE threat and per CPS component. It also depicts the associated initial global risk (without controls) and the residual global risk (with the optimal controls applied). These values have been calculated by employing Algorithm 1.

Each line of Table 5 represents one of the STRIDE threats. The first column represents the global initial risk (i.e., without any security controls in place) of the C-ES, as assessed by means of Algorithm 1. The second column represents each constituent CPS, and the third column the optimal set of security controls identified by means of Algorithm 2. Finally, the fourth column represents the residual risk (i.e., with the optimal set of security controls in place) of the C-ES, as assessed by applying again Algorithm 1 with the risks of each individual CPS updated according to the effectiveness of the applied controls.

### 6.3. Optimal Controls for the Remotely Controlled Ship

Remotely controlled vessels are equipped with CPSs that allow the control and operation of the vessel from the shore. Similarly with the autonomous vessel variant, the navigational CPSs of the remotely controlled ship are described by the directed graphs $G_{I}^{\prime }\left(V,E\right)$ and $G_{C}^{\prime }\left(V,E\right)$ in Figure 5 and Figure 6. The SCC is a critical component in this variant of the C-ES, since the control and monitoring of the vessel critically depends on the SCC’s normal operation. This is why the effect coefficients attain high values between systems that support the remote operations, such as the SCC, ANS, and ECDIS. All effect coefficients between the CPSs of the remotely controlled vessel are depicted in Table 6. Similarly to the case of the autonomous ship, the total effect coefficients have been calculated by means of Equation (13).

The security controls in the optimal set are selected from the initial list of available controls by applying the method described in Section 5. Table 7 depicts the optimal set of security controls per STRIDE threat and per CPS component. It also depicts the associated initial global risk (without controls) and the residual global risk (with the optimal controls applied). These values have been calculated in the same manner as the corresponding ones of the first C-ES variant.

### 6.4. Discussion

**Figure 7:**
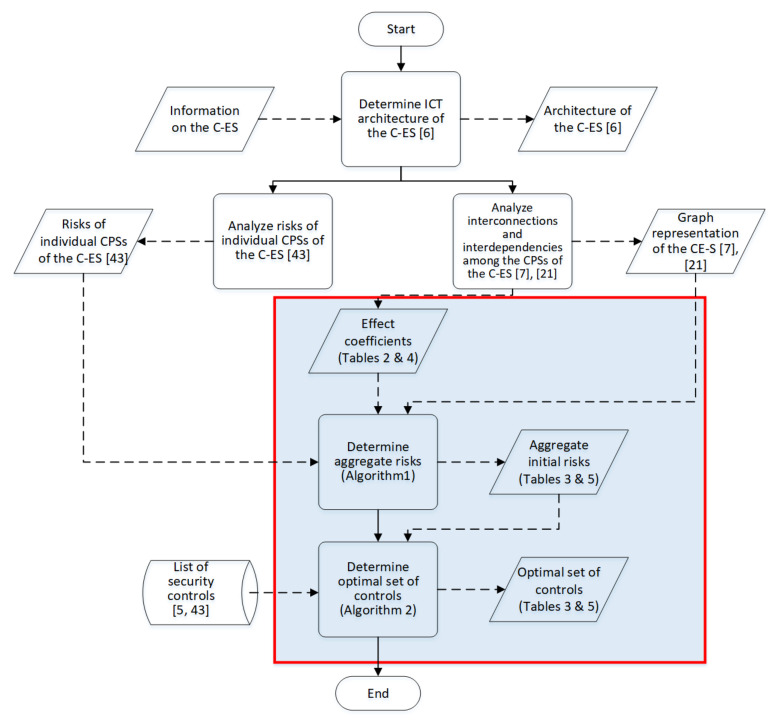
Overall process.

The overall process followed to carry out the case studies is depicted graphically in Figure 7. In this figure, rectangles represent processing steps, and skewed rectangles represent input/output; solid lines link processing steps, whilst dashed ones link input/output to processing steps. The shaded area delineates the content of this paper.

As can be seen in Table 5, in the case of the autonomous ship, twenty different security controls are recommended for application to seven of the ten navigational CPSs. The fact that these CPSs have been found in previous works [6,44] to be the most vulnerable onboard navigational systems, verifies the consistency of the proposed methods. Similarly, as can be seen in Table 7, twenty different security controls are recommended for application to six out of the ten navigational CPSs; again, these CPSs are the most vulnerable.

The optimal controls sets are different in the two variants of the C-ES. This reflects the difference in the level of autonomy of each variant: According to the IMO classification, the remotely controlled vessel lies at the second or third autonomy level, while the autonomous ship lies at the fourth level [52]. Different levels of autonomy mean different levels of interaction with humans and different levels of importance of the SCC in the ship’s operation, which, in turn, mean different levels of risk for the same threat.

The security controls that are recommended by any automated decision support method, including the methods proposed herein, need to be *re-considered*, *consolidated*, and *checked for applicability* by domain experts and stakeholders together. The proposed methods enable the execution of what-if scenarios, including by modifying the initial list of the available security controls, and/or by modifying parameters of the genetic algorithm.

## 7. Conclusions

The growing utilization of highly interconnected CPSs in critical domains increases the attack surface, making the infrastructure more vulnerable to cyber attacks. In this paper, we model a complex CPS as a digraph in which nodes represent sub-CPSs and in which edges represent information and control flows among these subsystems. By leveraging this model, we proposed a novel method for assessing the aggregate cybersecurity risk of large scale, complex CPSs comprising interconnected and interdependent components, by using risk measures of its individual components and the information and control flows among these components. Building upon this method, we proposed a novel method, based on evolutionary programming, for selecting a set of effective and efficient cybersecurity controls among those in an established knowledge base, that reduces the aggregate residual risk, while at the same time minimizing the cost. We then used both methods to select optimal sets of cybersecurity controls for the navigational systems of two instances of the C-ES, namely the remotely controlled ship and the autonomous ship. These sets lead to the definition of the cybersecurity architecture of such vessels. They have been found to be in line with previous results that identified the most vulnerable navigational CPSs of the C-ES, and to minimize the global residual risk. In the future, we intend to develop a software tool that will implement the proposed methods, and to use it to experientially examine the usability of the proposed approach with domain experts and stakeholders, in the C-ES and other critical application domains.

## Figures and Tables

**Table 1:** Criteria for determining the values of the DREAD (*D*amage, *R*eproducibility, *E*xploitability, *A*ffected, and *D*iscoverability) variables [22,44].

	High (3)	Medium (2)	Low (1)
**D**	The adversary is able to bypass security mechanisms; get administrator access; upload/modify the CPS content.	Leakage of confidential information of the CPSs (functions/source code); partial malfunction/disruption of the system.	Leakage of non-sensitive information; the attack is not possible to extend to other CPSs on-board.
**R**	The attack can be reproduced at anytime.	The adversary is able to reproduce the attack, but under specific risk conditions.	Although the attacker knows the CPS’s vulnerabilities/faults, they are unable to launch the attack.
**E**	The attack can be performed by a novice adversary, in a short time.	A skilled adversary may launch the attack.	The attack requires an extremely skilled person and in-depth knowledge of the targeted CPS.
**A**	All CPSs are affected.	Some users/systems, with non-default configuration are affected.	The attack affects only the targeted CPS.
**D**	The CPS’s vulnerabilities are well known, and the attacker is able to access the relevant information to exploit them.	The CPS’s vulnerabilities/faults are not well known and the adversary needs to access the CPS.	The threat has been identified, and the vulnerabilities have been patched.

**Table 2:** Impact values.

Impact										
	ANS	ASC	ASM	AIS	CA	ECDIS	SCC	RADAR	AP	VDR
**S**	2.5	3	2.5	2	2.5	2.5	2.5	2.5	2	2
**T**	2.5	2	1.28	2.5	2.5	2	2	2.5	2.5	2
**R**	2	2.5	1.5	2	1.5	1.5	1.5	2	1.5	1.5
**I**	2.5	2.5	2	2	1.5	3	1.5	1	2	2
**D**	2.5	2.5	2	2	2.5	3	2.5	2	2.5	2
**E**	3	3	1.5	2.5	1.5	3	1.5	2	2	2

**Table 3:** Likelihood values.

Likelihood										
	ANS	ASC	ASM	AIS	CA	ECDIS	SCC	RADAR	AP	VDR
**S**	1.33	1.33	2	2.66	1.33	2.32	1.66	2	1	1
**T**	1.33	2	1.28	2.33	1.66	2.33	1.33	1.66	1	1
**R**	1	1	1	2.66	1	1	1.33	1.33	1	1
**I**	1	1	1.33	2.66	1.33	1.66	1.33	1	1	1
**D**	1.33	1.66	2	2	1.33	2	1.66	2	1	1
**E**	1.33	1	1	1.33	1	1.66	1	1	1	1

**Table 4:** Effect coefficients—Autonomous ship.

	C-ES	AIS	ECDIS	VDR	ASM	RADAR	AP	CA	ANS	ASC
**C-ES**	0	0	0	0	0	0	0	0	0	0
**ANS**	0.208	0.208	0.208	0.208	0.208	0.166	0.208	0.208	0	0
**ASC**	0.055	0.055	0.055	0.055	0.055	0	0.055	0.055	0.055	0
**ASM**	0.321	0.071	0.071	0	0	0	0.071	0.321	0.071	0.071
**AIS**	0.041	0	0.041	0.041	0.041	0.041	0.041	0.041	0.041	0.041
**CA**	0.211	0.211	0.045	0.045	0.045	0.045	0.045	0	0.211	0.045
**ECDIS**	0.05	0.05	0	0.05	0.05	0.05	0.05	0.05	0.05	0.05
**RADAR**	0,055	0	0.055	0.055	0	0	0.055	0.055	0.555	0
**AP**	0,045	0.045	0.045	0	0	0.045	0	0.045	0.045	0.045
**VDR**	0,062	0.062	0.062	0	0	0.062	0	0	0.062	0.062

**Table 5:** Optimal controls—Autonomous ship.

Threat	Initial Risk	Component	Controls	Residual Risk
Spoofing	1.651	ECDIS	Time Stamps (AU-8)	0.964
		ASM	Unsuccessful Logon Attempts (AC-7)	
		AIS	Remote Access (AC-17)	
		Radar	Security Assessments (CA-2)	
Tampering	1.615	AIS	Information Input Restrictions (SI-9)	1.087
		Radar	Tamper Protection (PE-3(5))	
		CA	Tamper Protection (PE-3(5))	
		ECDIS	Port and I/O Device Access (SC-41)	
		ASC	Tamper Protection (PE-3(5))	
Repudiation	1.555	Radar	Device Identification and Authentication (IA-3)	0.725
		AIS	Information System Component Inventory (CM-8 (4))	
Information Disclosure	1.629	AIS	Cryptographic Protection (SC-13)	0.89
		CA	Information System Component Inventory (CM-8 (4))	
		ECDIS	Protection of Information at Rest (SC-28)	
Denial of Service	1.373	AIS	Denial of Service Protection (SC-5)	0.89
		Radar	Fail-Safe Procedures (SI-17)	
		CA	Denial of Service Protection (SC-5)	
		ANS	Fail-Safe Procedures (SI-17)	
		ASC	Power Equipment and Cabling (PE-9)	
		ECDIS	Device Identification and Authentication (IA-3)	
		ASM	Fail-Safe Procedures (SI-17)	
Elevation of Privileges	1.129	ANS	Device Identification and Authentication (IA-3)	0.725
		AIS	Internal System Connections (CA-9)	
		ECDIS	Unsuccessful Logon Attempts (AC-7)	

**Table 6:** Effect coefficients—Remotely controlled ship.

	C-ES	AIS	ECDIS	VDR	ASM	RADAR	AP	CA	ANS	SCC
**C-ES**	0	0	0	0	0	0	0	0	0	0
**ANS**	0.208	0.208	0.208	0.208	0.208	0.106	0.208	0.208	0	0.208
**SCC**	0.75	0.5	0.75	0.5	0.5	0	0.75	0.5	0.75	0
**ASM**	0	0.071	0.071	0	0	0	0.071	0.321	0.071	0
**AIS**	0.041	0	0.041	0.041	0.041	0.041	0.041	0.041	0.041	0.041
**CA**	0	0.295	0.045	0.045	0.045	0.045	0.045	0	0.295	0
**ECDIS**	0.05	0.05	0	0	0.05	0.05	0.05	0.05	0.05	0.05
**RADAR**	0.166	0.166	0.166	0.166	0	0	0.166	0.166	0.666	0.166
**AP**	0.045	0.045	0.045	0	0.045	0.045	0	0.045	0.045	0
**VDR**	0	0.062	0.062	0	0	0.062	0	0	0.062	0

**Table 7:** Optimal controls–Remotely controlled ship.

Threat	Initial Risk	Component	Controls	Residual Risk
Spoofing	1.952	SCC	Monitoring Physical Access (PE-6 (1))	1.663
		ASM	Unsuccessful Logon Attempts (AC-7)	
		AIS	Remote Access (AC-17)	
		Radar	Security Assessments (CA-2)	
Tampering	1.663	ECDIS	Device Identification and Authentication (IA-3)	1.04
		ANS	Port and I/O Device Access (SC-41)	
		Radar	Tamper Protection (PE-3(5))	
		CA	Tamper Protection (PE-3(5))	
		SCC	Physical Access Control (PE-3)	
		AIS	Information Input Validation (SI-10)	
Repudiation	1.828	AIS	Device Identification and Authentication (IA-3)	0.875
		Radar	Security Assessments (CA-2)	
		SCC	Non-repudiation (AU-10)	
Information Disclosure	1.828	AIS	Cryptographic Protection (SC-13)	1.47
		SCC	Information System Component Inventory (CM-8 (4))	
Denial of Service	1.622	ECDIS	Internal System Connections (CA-9)	0.99
		AIS	Information System Backup (CP-9 (1), (2), (3), (5))	
		CA	Denial of Service Protection (SC-5)	
		SCC	Denial of Service Protection (SC-5)	
		Radar	Security Assessments (CA-2)	
		ANS	Emergency Shutoff (PE-10)	
		ASM	Fail-Safe Procedures (SI-17)	
Elevation of Privileges	1.205	ANS	Device Identification and Authentication (IA-3)	0.875
		AIS	Internal System Connections (CA-9)	
		ECDIS	Unsuccessful Logon Attempts (AC-7)	
